# Temperature-controlled spectral tuning of full-color carbon dots and their strongly fluorescent solid-state polymer composites for light-emitting diodes†

**DOI:** 10.1039/c8na00329g

**Published:** 2019-01-17

**Authors:** Tantan Hu, Zhuoqi Wen, Chan Wang, Tiju Thomas, Chuanxi Wang, Qijun Song, Minghui Yang

**Affiliations:** Institute of New Energy Technology, Ningbo Institute of Industrial Technology, Chinese Academy of Sciences Ningbo 315201 P. R. China wangcx@nimte.ac.cn myang@nimte.ac.cn; Key Laboratory of Synthetic and Biological Colloids, Ministry of Education, International Joint Research Center for Photoresponsive Molecules and Materials, School of Chemical & Material Engineering, Jiangnan University Wuxi 214122 P. R. China wangchan@jiangnan.edu.cn.; Department of Metallurgical and Materials Engineering, Indian Institute of Technology Madras Chennai 600036 Tamil Nadu India

## Abstract

The development of full-color/white carbon-dot-based light-emitting diodes (LEDs) has been achieved, which show promising applications in full-color and flexible displays, backlights, and novel lighting sources. The gram-level synthesis of these full-color carbon dots (CDs) from citric acid by controlling the temperature has been achieved. By increasing the temperature from 120 to 180 °C, two, four, and six light-emitting CDs can be obtained, for which the emission wavelength shifts from 440 to 585 nm. This result reveals that temperature has a huge impact on the evolution of surface states, that is, increasing the temperature brings about enhanced surface functionalization and passivation, resulting in a red shift of the emission wavelength and enhancement of quantum yield. Then, full-color CDs/polymer composite phosphors are fabricated for efficient phosphor-based LED devices with quench-resistant solid-state fluorescence. By regulating the proportion of various CDs/polymer phosphors, white LEDs are realized with Commission Internationale de L'Eclairage coordinates of (0.32, 0.33) and a color rendering index of 82.7. The as-prepared CD-based full/white color LEDs can prove to be promising candidates for alternative light sources.

## Abstract 2

Temperature-controlled, scalable synthesis of CA-based full-color CDs and subsequent fabrication of white-LEDs with CIE coordinates of (0.32, 0.33) and a CRI of 82.7.

## Graphical abstract

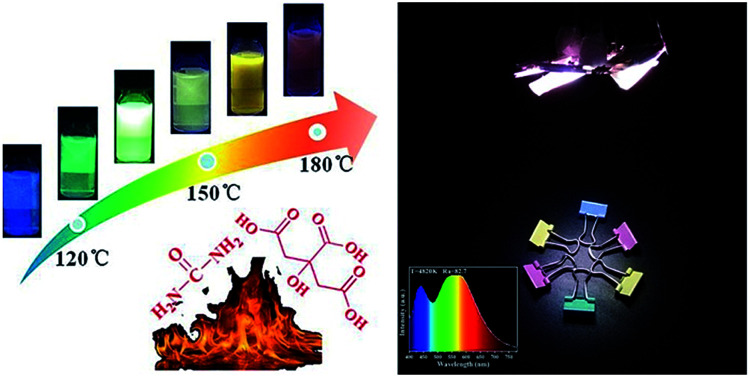

## Introduction

Solid-state light emitting diodes (LEDs), especially white LEDs, have several desirable features such as long lifetime, low power consumption, compact size and high photoelectric conversion efficiency. Hence it is promising for traffic lighting, decorative lighting, display lighting *etc.*^1–6^ Attempts are ongoing to make white LEDs more relevant and appealing for indoor applications too. To date, significant progress has been made in producing white LEDs using a combination of UV/blue LED chips and multi-color phosphors. Very often the phosphors are based on rare-earth compounds or/and semiconductor quantum dots (QDs).^7–10^ However, rare earth-based phosphors have a high-cost associated with them and the synthesis and processing conditions required limit their practical and large scale use. Cd^2+^/Pb^2+^-based QD phosphors, while being very promising, show inherent toxicity, which hinders their practical application in next-generation luminophores for future LED technologies. Recently, multi-color carbon dots (CDs) have emerged as promising candidates for LED applications owing to their stability, low toxicity, and ease of preparation.^11–14^ Moreover, white light can be obtained by regulating the proportion of various CDs that emit different colors.^15–18^ Hence, CDs are great candidates for alternative multi-color phosphors.^19–23^ However challenges remain in the area; the major current concerns are to do with stable long-wavelength emission of CDs and prevention of aggregation-caused quenching in their solid state.

It has been reported that CDs with long wavelength emission can be achieved using the solvothermal treatment of aromatic compounds. However the choice and need of special carbon precursors (phenylenediamine, polythiophene derivatives, azido imidazole *etc.*) limit their wide application.^18,24–28^ Therefore, the use of a common carbon source (say citric acid) for preparing CDs with long wavelength emission is pivotal for contemporary technology. As a common reagent, citric acid (CA) plays an important role in the synthesis of CDs through several reported bottom-up methods.^29–32^ Since a large number of hydrophilic groups exist in citric acid, CA-based-CDs have good hydrophilicity, and show high photoluminescence quantum yield (PLQY). Hence, it is significant to study the luminescent properties of CA-based-CDs. Zhu *et al.* prepared several CA-based CDs and found that an important PL center in all of them is a molecular complex, which has covalently bonded carbon cores.^31^ However, these CA-based-CDs are limited in their application due to their use in short-wavelength emission. Some recent studies have been done on the preparation of CA-based-CDs with long wavelength emission. Tian *et al.* obtained full-color emissive CDs from CA and urea by employing three different solvents.^4^ Katerina Hola and co-workers prepared full-color CDs with controllable fluorescence from CA and urea in formamide.^13^ Sun *et al.* synthesized multiple-color-emissive CDs by regulating the thermal-pyrolysis temperature and ratio of reactants (CA and urea).^14^ However, up to now, no definite luminescence/spectral characteristic-controllable synthesis parameter has been explored in detail. This is obviously essential for the fabrication of LEDs based on CA-derived full-color CDs.

It may be noted that immobilizing CDs in a solid matrix is a suitable way to overcome their emission quenching in the solid state.^33–37^ For example, Zhou *et al.* prepared CDs@BaSO_4_ hybrid phosphors by assembling Ba^2+^ and SO_4_^2−^ onto the surface of CDs through electrostatic attraction, in which the CDs were the luminescence center.^5^ Shao and co-workers mixed polymer CDs with starch to fabricate multi-color light-emitting phosphors.^38^ However, there are very few reports about CA-based full-color CD solid state phosphors which are relevant from the standpoint of scalable production. Due to the above reasons, in this study, some novel full-color CD-based solid state phosphors for LEDs are prepared. These full-color CDs are synthesized by regulating the pyrolysis temperature using CA as the carbon source. Two, four, and six light-emitting CA-based CDs are obtained readily by increasing the reaction temperature from 120 to 180 °C. In addition, the as-prepared CDs emit bright and stable full-color fluorescence from blue to orange-red. Then, the luminescent and controllable synthesis mechanism for these CA-based full-color CDs is proposed. It is revealed that temperature plays a key role in surface passivation and heteroatom-doping, and hence in the formation of surface states, which can make the emission wavelength red-shift and PL quantum yield (PLQY) increase. Thus, a route for the temperature-controlled and large-scale synthesis of CA-based full-color CDs is reported. The CDs were immobilized in a solid polymer network, which can prevent their aggregation and form CA-based full-color CDs/polymer phosphors with bright fluorescence. Full/white color LEDs are prepared using these phosphors, and their performance is compared and found to be comparable with some of the best results reported so far. Therefore, the as-prepared LEDs from CA-based full-color CDs can prove to be promising candidates for alternative light sources.

## Experimental

### Materials

Urea (MW = 60.06) and methanol were acquired from Sinopharm Chemical Reagent Co., Ltd. (China). *N*,*N*-Dimethylformamide (DMF) and dichloromethane were obtained from Shanghai Titan Scientific Co., Ltd. Citric acid monohydrate (CA, MW = 210.14) was obtained from Tianjin Bodi Chemical Co., Ltd (China). All other chemicals were of analytical grade and purchased from Sinopharm Chemical Reagent Co., Ltd. (Shanghai, China). All materials were used as obtained without further purification. Deionized water was used in all the experiments.

### Synthesis of CA-based full color CDs

CA-based full color CDs are synthesized through a simple one-step solvothermal treatment of CA and urea. The specific synthesis steps are described below. First, 1.0 g of CA and 2.0 g of urea are dissolved in 10 mL of DMF solution; then, the mixture is transferred into a Teflon-lined stainless-steel autoclave, and heated at 120, 150 and 180 °C for 6 h. After cooling to room temperature, the acquired solution is purified *via* silica column chromatography with a mixture of dichloromethane and methanol as the eluent. Finally, the obtained multi-color CD samples are redispersed in water and freeze-dried to form a powder for further use.

### Preparation of full-color/white emission CD/PVA films

PVA solutions with a concentration of 15% are prepared by dissolving 6 g into 34 mL deionized water at 90 °C for 10 h. Then 10 mg blue, green, yellow, and orange-red light emitting CD powders are added to the 15% PVA solution (10 mL) while stirring the mixture continuously. For the white light-emitting CD/PVA solution, 2 mg B-CD, 2 mg G-CD, and 3 mg O-CD powders are mixed with 15% PVA solution (10 mL) under stirring. Finally, the CD/PVA solution is coated on a quartz plate, and naturally cured at room temperature to obtain CD/PVA films with full-color emission.

### Fabrication of LEDs based on CD/polymer composites

Commercially available GaN LED chips (the emission centered at 365 nm) without phosphor coating are purchased from Advanced Optoelectronic Technology Inc. Then, the blue light-emitting CD/PVA film is fastened to the top of the GaN LED chip to achieve the fabrication of blue LEDs. The green LEDs, yellow LEDs, orange-red LEDs and white LEDs are also prepared using the above method.

### Calculation of the absolute photoluminescence quantum yield

The quantum yield was measured using a time-resolved and steady state fluorescence spectrometer, Horiba Jobin Yvon Fluoromax 4C-L (France). The absolute quantum yield (QY) can be calculated using the following equation:^50^
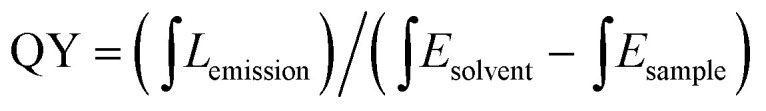
where QY is the absolute quantum yield, *L*_emission_ is the photon number of the FL emission of CDs, and *E*_sample_ and *E*_solvent_ are the photon numbers of excitation light used for excitation of CDs and the solvent (DI water), respectively.

### Characterization

A UV-vis spectrophotometer (Hitachi U-3900, Japan) was used to obtain UV-vis absorption spectra. Photoluminescence (PL) spectra were recorded on a Horiba Jobin Yvon Fluoromax 4C-L (France) spectrophotometer. Nanosecond fluorescence lifetime experiments were performed using a time-correlated single-photon counting (TCSPC) system, and the results were recorded using a Horiba Jobin Yvon Fluoromax 4C-L (France) spectrophotometer. A 370 and 458 nm picosecond diode laser was used to excite the samples. Transmission electron microscopy (TEM) images were studied using a Tecnai GI F20 U-TWIN with an accelerating voltage of 200 kV. X-ray photoelectron spectra were recorded using an AXIS ULTRA DLD (Shimadzu, Japan) spectrometer with Mg Kα excitation (1253.6 eV). Binding energy calibration was based on C 1s at 284.6 eV. A Nicolet 6700 FTIR spectrophotometer was used to collect Fourier transform infrared (FTIR) spectra ranging from 4000 to 400 cm^−1^. The electroluminescence (EL) spectra of full-color/white LEDs were recorded by combining a Spectra scan PR-650 spectrophotometer with an integrating sphere and a computer-controlled direct current power under ambient conditions at room temperature. The absolute quantum yield was measured on a QE-2100 (Japanese amnesty) with an integrating sphere.

## Results and discussion

**Fig. 1:**
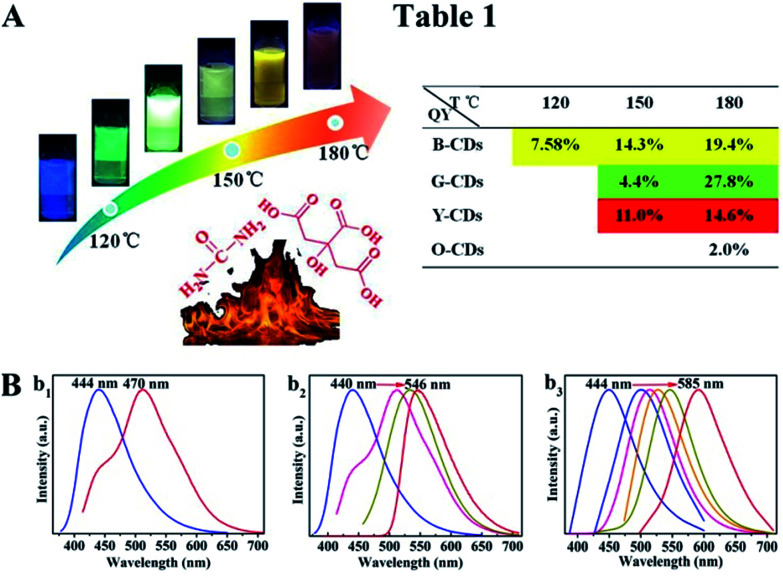
(A) The schematic diagram of temperature-controlled and large-scale synthesis of CA-based full-color CDs. Table 1 contains the relative quantum yield data analysis of the resultant CDs at different temperatures. (B) Corresponding PL spectra of CA-based CDs prepared at different reaction temperatures: (b_1_) 120 °C; (b_2_) 150 °C; (b_3_) 180 °C, respectively.

The synthesis route of citric acid (CA)-based full-color CDs is shown in Fig. 1A. CA and urea are selected as the single carbon precursor and the nitrogen source, respectively. CA, a common carbon source, plays an important role in the synthesis of CDs with high PLQY, and nitrogen-doping can make the emission wavelength of CDs significantly red-shifted.^30,39–41^ More importantly, experimental conditions also make a dramatic difference to the PL properties, especially the reaction temperature, which plays an important role in achieving the controllable synthesis of CDs with different emission colors.^14^ Hence, light-emitting CDs that have varying spectral properties can be obtained by varying the reaction temperature as shown in Fig. 1A. By increasing the temperature from 120 to 180 °C, full-color CA-based CDs from blue to orange-red can be prepared (Fig. 1A). When the temperature is 120 °C, two emission peaks/colors of CDs in short wavelength can be obtained; the wavelengths observed are 444 and 470 nm, respectively (Fig. 1b_1_). Increasing the temperature to 150 °C results in emission that is discernibly red-shifted. In these CDs, four color-emitting systems (blue to yellow) can be prepared; however, the emission wavelength is limited to a maximum of ∼546 nm in this sample (Fig. 1b_2_). Furthermore, increasing the temperature up to 180 °C results in full color-emitting CDs (Fig. 1b_3_). Orange-red light-emitting CDs with optimal emission at 585 nm are observed too (Fig. 1b_3_). Table 1 exhibits the PLQYs of the as-prepared CDs, synthesized at different temperatures. It is interesting that the PLQYs of mono color-emitting CDs increase with increasing pyrolysis temperatures (Table 1). For instant, the PLQY of blue light-emitting CDs (referred to as B-CDs) increases from 7.6 to 19.4% (using quinine sulfate as the reference, QY = 54%) when increasing the temperature from 120 to 180 °C (Table 1).

**Fig. 2:**
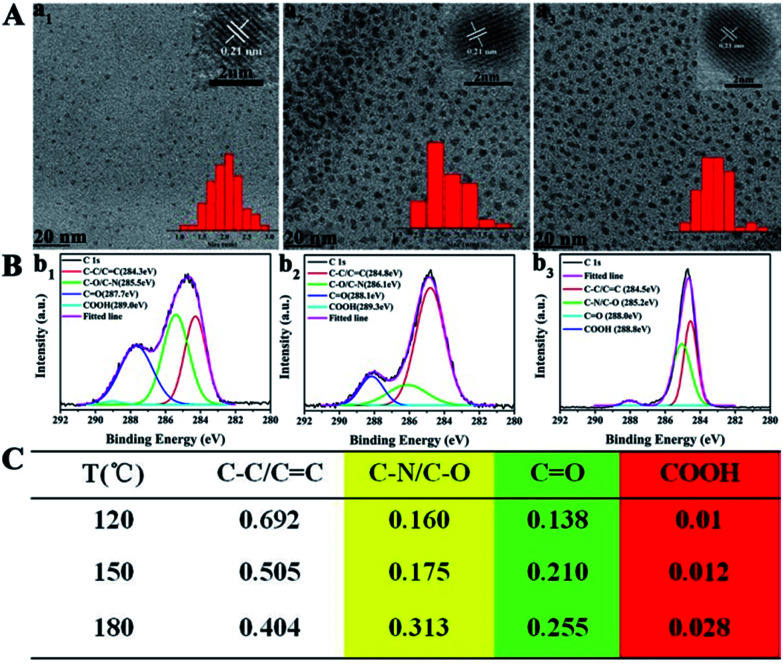
(A) TEM images of blue light-emitting CDs prepared at temperatures of 120 (a_1_), 150 (a_2_), and 180 °C (a_3_), respectively. (B) High-resolution XPS C 1s spectra of blue light-emitting CDs prepared at temperatures of 120 (b_1_), 150 (b_2_), and 180 °C (b_3_), and each band is deconvoluted following the literature. (C) XPS data analyses of the C 1s spectra of blue light-emitting CDs.

To better understand the role of temperature in the controlled synthesis, a variety of characterization studies are carried out. First of all, the blue light-emitting CDs (referred to as B-CDs) prepared at 120, 150, and 180 °C are investigated and they show similar emission (Fig. S1†); however, the PLQYs vary (Table 1). Besides, the emission of all blue light-emitting CDs shows excitation wavelength dependence (Fig. S2†), indicating that the luminescence center on its surface is not clear.^42,43^ Moreover, all the B-CDs show good dispersion (Fig. 2A) and uniform size distribution (inset in Fig. 2A). As the reaction temperature increases, the average size of the CDs increases from 2.0 to 2.8 nm (Fig. 2A). High-resolution TEM images (inset in Fig. 2A) provide clear evidence that all the B-CDs possess the same lattice spacing of 0.21 nm, attributed to the *d* spacing of the graphene (100) planes.^44^ These results also confirm that all the B-CDs have similar size of isolated sp^2^ domains.^14^ XPS and FTIR are used to characterize their surface state. FTIR spectra (Fig. S3†) show that the three B-CDs possess similar functional groups, such as N–H (3450 cm^−1^), O–H (3200 cm^−1^), C=O (1728 cm^−1^), C=N (1673 cm^−1^), C=C (1574 cm^−1^), C–N (1475 cm^−1^), *etc.*, while the intensity of each characteristic peak is quite different.^12,45^ With the increase in pyrolysis temperature, the peak intensity of functional groups increases, indicating that the degree of surface functionalization deepens. All the B-CDs contain C, O, and N elements (Fig. S4†).^13,46^ The difference among them is that the O/C ratio increases, while the N to C ratio remains constant. And their high-resolution XPS C 1s band can be deconvoluted into four main components: sp^2^ carbons (C=C/C=N), sp^3^ carbons (C–O/C–N), carbonyl carbons (C=O), and carboxyl carbons (COOH) (Fig. 2B).^12,47^ XPS analysis (Fig. 2C) reveals that with the increase of pyrolysis temperature, the C–N/C–O content of the three B-CDs increases significantly, while the content of COOH is almost unchanged, indicating that the increasing temperature will accelerate the escape of N and O elements from the carbon nucleus. Consequently, increasing pyrolysis temperature results in a greater degree of surface functionalization, which is why the PLQY of B-CDs increases from 7.6 to 19.4%.

**Fig. 3:**
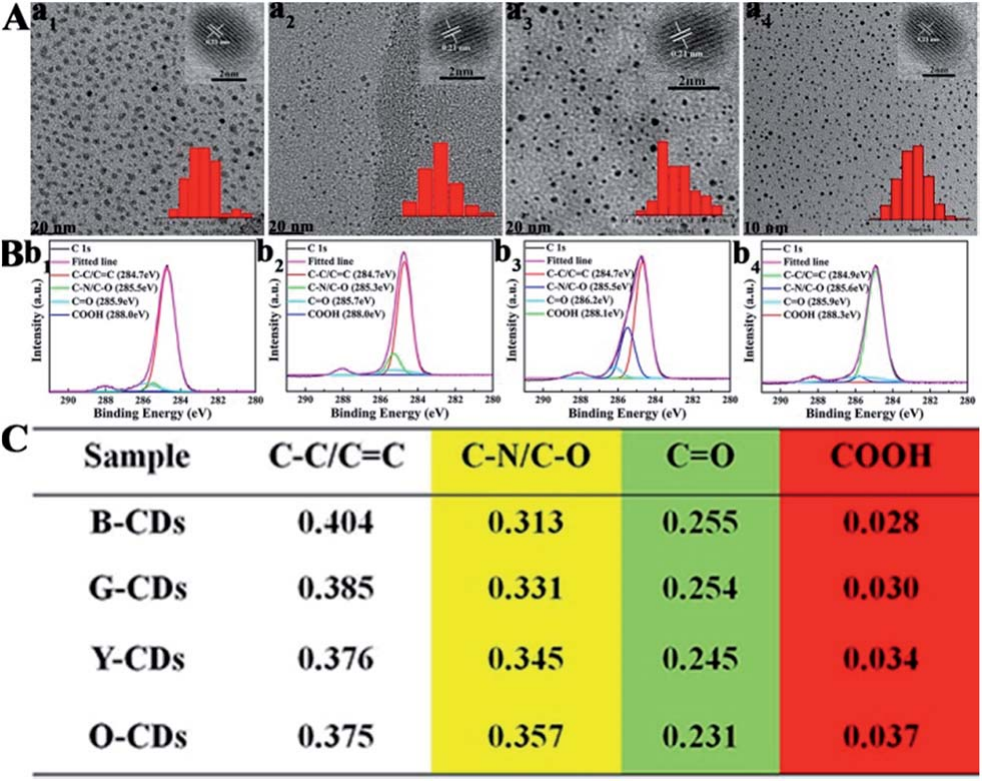
(A) TEM images of blue (a_1_), green (a_2_), yellow (a_3_), and orange-red (a_4_) light-emitting CDs prepared at 180 °C. (B) High-resolution XPS C 1s spectra of blue (b_1_), green (b_2_), yellow (b_3_) and orange-red (b_4_) light-emitting CDs prepared at 180 °C, and each band was deconvoluted following the literature. (C) XPS data analyses of the C 1s spectra of the four color-emitting CDs.

Secondly, blue, green, yellow, and orange-red light-emitting CDs (referred to as B-, G-, Y-, and O-CDs) prepared at 180 °C are further studied. Their emission wavelengths are 444, 510, 550 and 585 nm (Fig. S5†), and the PLQYs are 19.4, 27.8, 14.6, and 2.0% (Table 1), respectively. The UV-vis absorption spectra (Fig. S5†) reveal that the four CDs exhibit main absorption bands at about 360, 420, 460 and 460 nm, respectively, which are attributed to the n–π* transitions of the C=O/C=N bond.^31^ Moreover, the excitation peaks of the four CDs (Fig. S5†) are at 365, 450, 420 and 400 nm, respectively; this correlates with their absorption bands in this region.^12^ In addition, with a change in the excitation wavelength, the emission wavelengths of B- and G-CDs shift, since there are evidently different emission sites on their surface. In contrast, the emission wavelengths of Y- and O-CDs show no shift (Fig. S6†). The TEM and HRTEM images of the four CDs are displayed in Fig. 3A. Their average sizes are 2.5, 1.7, 1.8, and 3.5 nm with the same lattice structure (*d* spacing ∼ 0.21 nm), corresponding to the (100) in-plane lattice of graphene. The FTIR spectra (Fig. S7†) show that stretching vibrations of N–H (3450 cm^−1^), O–H (3200 cm^−1^), C=O (1728 cm^−1^), C=N (1673 cm^−1^), C=C (1574 cm^−1^), and C–N (1475 cm^−1^) are present in all the four CDs. Interestingly C=O and N–H stretching vibration trends indicate a transition from B-CDs to O-CDs. Fig. S8† shows that the four CDs all consist of the same elements (C, N, and O) on their surfaces, and the ratio of N/O increases from B-CDs to O-CDs (Fig. S9†). These results confirm the increased degree of oxidation and the substitution of hydroxyl groups by amino groups, which leads to the PL emission red-shift. The high-resolution C 1s spectra (Fig. 3B) of all the CDs can be deconvoluted into four main components: sp^2^ carbons (C=C/C=N), sp^3^ carbons (C–O/C–N), carbonyl carbons (C=O), and carboxyl carbons (COOH). Further analysis of XPS data reveals that the content of –COOH and C–N increases, while the content of C=O decreases (Fig. 3C). This is a hint that the degree of surface passivation increases and the content of nitrogen-doping increases from B-CDs to O-CDs.^12^ The band-gap energy of the four CDs gradually decreases from 3.37 to 2.31 eV from B-CDs to O-CDs (Fig. S10 and Table S1†), which is close to the theoretical value.

The time-resolved fluorescence spectra show the four CDs with exponential decay and with average delayed fluorescence (DF) lifetimes of 5.73, 6.43, 5.87, and 3.90 ns (Fig. S11 and Table S1†), respectively. As reports showed, the two radiative lifetimes *τ*_1_ and *τ*_2_ might be assigned to the intrinsic recombination of initially populated core states and surface states, respectively. Based on the fitted values in Table S1,†*τ*_2_ (long-lived component) is the dominant factor in the radiative lifetime, indicating that the fluorescence decay kinetics of these CDs was mainly caused by their surface states.^19^ All the results testify that each kind of CD has a specific absorption structure and luminescent center, and PL behavior of each of them is obviously derived from absorption properties in the low-energy region. The main reason for the red-shift of emission wavelength with prolonged isothermal treatment has to do with enhanced surface passivation and increased nitrogen doping content.

**Fig. 4:**
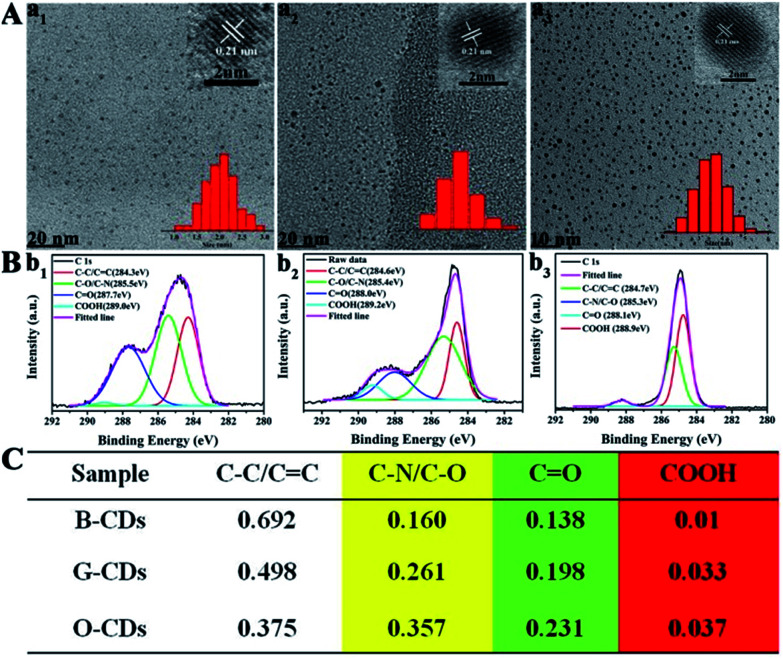
(A) TEM images of blue, green, and orange-red light-emitting CDs prepared at temperatures of 120 (a_1_), 150 (a_2_), and 180 °C (a_3_), respectively. (B) High-resolution XPS C 1s spectra of blue, green, and orange-red light-emitting CDs prepared at temperatures of 120 (b_1_), 150 (b_2_), and 180 °C (b_3_), and each band is deconvoluted following the literature. (C) XPS data analyses of the C 1s spectra of these three color-emitting CDs.

Finally, blue (120 °C), green (150 °C), and orange-red (180 °C) light-emitting CDs (referred to as B-CDs_120_, G-CDs_150_, and O-CDs_180_) are selected for further comparison. As illustrated in Fig. S12 and S13,† the B-CDs_120_ and G-CDs_150_ show typical excitation-wavelength dependent emission with optimal wavelengths at 444 and 513 nm, while O-CDs_180_ show an emission peak centered at 585 nm without excitation wavelength dependence. Fig. 4A confirms that all the CDs exhibit homogeneous dispersion with average sizes of 2.0, 1.8, and 3.5 nm, and possess the same lattice spacing of 0.21 nm. Similar functional groups are present on the surface of B-CDs_120_, G-CDs_150_, and O-CDs_180_. The groups present include N–H (3450 cm^−1^), O–H (3200 cm^−1^), C=O (1728 cm^−1^), C=N (1673 cm^−1^), C=C (1574 cm^−1^), C–N (1475 cm^−1^), *etc.*, as shown in the FTIR spectra (Fig. S14†). However, the intensity of the typical C=O and N–H stretching vibrations increases, while the strength of O–H stretching vibrations decreases, as one goes from B-CDs_120_ to O-CDs_180_. The full XPS spectra of all the CDs presented in Fig. S15† show three typical peaks: C 1s, O 1s, and N 1s. In the high-resolution spectra (Fig. 4B), the C 1s band can be deconvoluted into four peaks: sp^2^ carbons (C=C/C=N), sp^3^ carbons (C–O/C–N), carbonyl carbons (C=O), and carboxyl carbons (COOH). As revealed in the detailed XPS analysis (Fig. 4C), with the increase of pyrolysis temperature, the XPS intensity of COOH and C–N increases significantly, indicating that the degree of surface passivation increases and the content of nitrogen-doping increases. To sum up, the higher degree of surface passivation and increased nitrogen doping content of the CDs with increasing pyrolysis temperature can account for the red-shift of emission wavelength continuously from 444 to 585 nm.

**Fig. 5:**
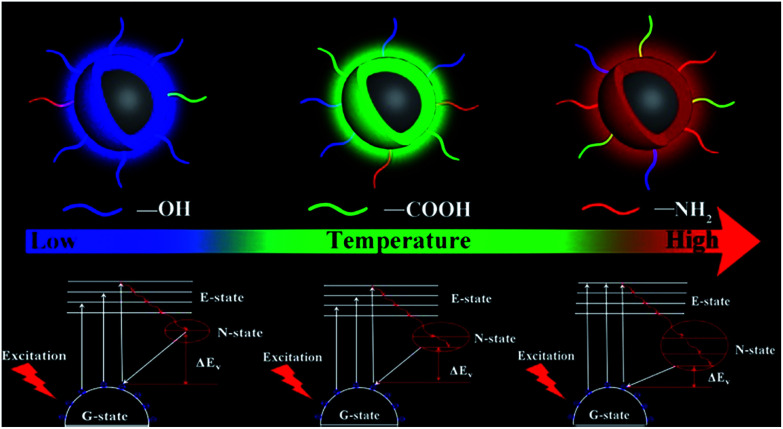
Schematic diagram of the luminescent and controllable synthesis mechanism.

The reasonable luminescent and controllable synthesis mechanism is shown in Fig. 5. This mechanism is mainly composed of two parts. The first part is that the increased temperature will accelerate the escape of N and O elements from the carbon nucleus, resulting in the improved surface functionalization. This is the reason for the increase in PLQYs for the same light-emitting/monochrome CDs. The other one is that the increasing temperature causes the higher degree of oxidation, which is conducive to surface passivation. And the enhanced surface passivation can not only make the emission wavelength significantly shift from the blue to the orange-red region, but also make surface emission sites uniform. Furthermore, nitrogen-doping can produce a new energy level, called the N-state.^39^ The introduction of a new energy level can significantly reduce the energy band gap. With the increase of nitrogen-doping content, this single energy level will be split into more energy levels, which further reduces the energy band gap. The synergistic effect of the two parts can make the emission wavelength significantly red shift and PLQY increase. Thus, based on this clear temperature-controlled synthesis and luminescent mechanism, the gram-level synthesis of CA-based full-color CDs was achieved by this simple method.

The controllable and large-scale synthesis of CA-based full-color CDs is achieved, and they have many advantages, such as bright fluorescence, low price, high stability, low toxicity, and easy preparation. Similar to many fluorescent nanomaterials, when these CDs are in their solid state, the fluorescence intensity is greatly reduced due to the aggregation induced quenching effect (Fig. S16A†).^34,38^ However, after mixing them with a polymer (PVP), full-color CD/PVP powder with strong solid-state fluorescence is obtained (Fig. S16B†). The polymer matrix results in uniform distribution of CDs, and thus would overcome the aggregation-induced quenching effect (Fig. S17†). Furthermore, full-color LED devices are fabricated using CD/PVP solid-state phosphors as emitting layers, which showed four-color solid state light emitting, respectively (Fig. S16C†).

**Fig. 6:**
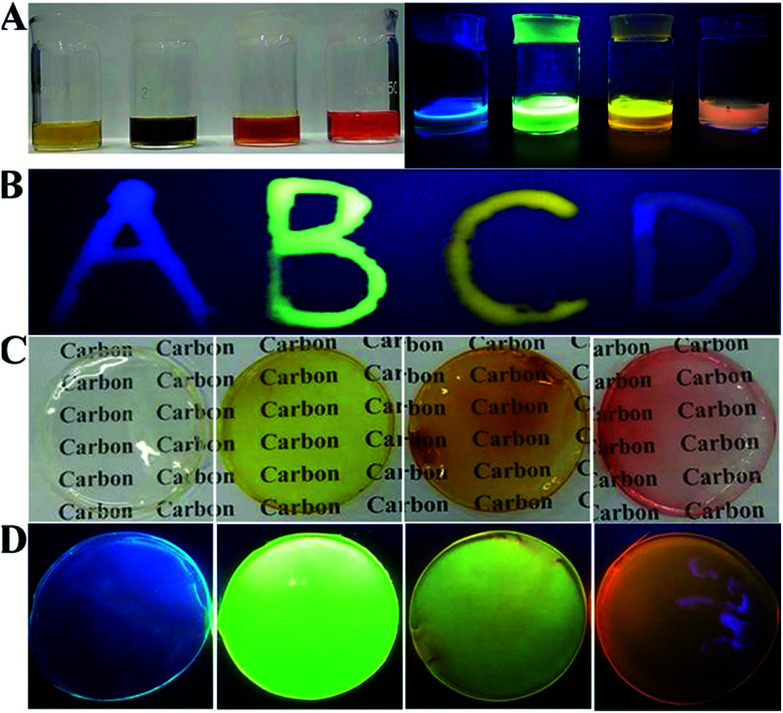
(A) Pictures of B-, G-, Y-, and O-CD/PVA solutions under daylight and UV light. (B) The images of four characters under UV light, with B-, G-, Y-, and O-CD/PVA solutions as ink. (C) The pictures of B-, G-, Y-, and O-CD/PVA films under daylight. (D) The pictures of B-, G-, Y-, and O-CD/PVA films under UV light.

The polymer PVA is selected for preparing full-color CD-based solid-state phosphors, since PVA has pure surface conformation, environmental friendliness, low cost and excellent hydrophilicity. Moreover, the initial fluorescence properties of nanomaterials can be maintained in the polymer network.^33,48^ Similar to CDs/PVP, the composite solution of PVA and CDs shows quench-resistant fluorescence (Fig. 6A).^35,38,49^ It has high viscosity and can be used to form fluorescent ink to form various patterns, which show bright solid-state fluorescence at room temperature (Fig. 6B). Then, CD/PVA films were prepared, and they showed great performance on transmission of light and ductility (Fig. 6C). The PL spectra of CD/PVA films show their corresponding emission wavelengths centred at 444, 520, 550 and 585 nm, respectively (Fig. S18†). Moreover, when the CD/PVA films are exposed to UV light, they emit bright fluorescence with PLQYs of 5.3%, 12.4%, 8.9%, and 6.9% for blue, green, yellow, and orange-red emission (Fig. 6D and Table S3†). The PL excitation and emission spectra of the CD/PVA solutions and films are identical to that of a CD solution (Fig. S19 and S20†), indicating that the fundamental emissive properties of CDs in PVA are well preserved. The consistency of FTIR results also verifies the above conclusion (Fig. S21†). The TEM image (Fig. S22†) demonstrates that the distribution of CDs in the PVA films is homogeneous. All the DF spectra for the four CD/PVA films are secondary exponential with average DF lifetimes of 3.85, 7.77, 4.88, and 2.91 ns (Fig. S23 and Table S3†), respectively. Furthermore, the stability of the CDs and CD/PVA films was tested in terms of photostability and thermal stability, respectively. The photostability of the CDs and CD/PVA films was studied at different exposure times under 365 nm UV light irradiation, respectively. The results are shown in Fig. S24A.† After 10 h of 365 nm UV light irradiation, for G-, Y-, and O-CDs, 15%, 20% and 21% loss was recorded, respectively. A slight increase of ∼10% was observed in the PL intensity of B-CDs. The same trend of change also appears in CD/PVA films. There are only minor changes observed, with >80% of the initial PL intensity maintained (Fig. S24B†). It is worth noting that after the combination of CDs with PVA, the photostability of CD/PVA films is improved, attributed to the positive influence of polymer on CDs. The FL signals of the CDs and CD/PVA films were also recorded at different temperatures varying from 25 to 120 °C, respectively. As shown in Fig. S25A,† for B-, G-, and Y-CDs, after 1 h of thermal treatment in the temperature range from 25 to 120 °C, there is a minor PL intensity loss, and about 90% of the PL intensity was preserved. However, for O-CDs, the increase in temperature made the PL intensity sharply decrease. When the temperature was 120 °C, a 60% loss could be observed, indicating that O-CDs possess temperature-responsive properties. The change in PL intensity of CD/PVA films is shown in Fig. S25B.† There are no major changes in the relative PL intensity of B-, G-, and Y-CD/PVA films, while a 30% loss was recorded for O-CD/PVA films. Compared with the 60% loss of PL intensity for O-CDs, the thermal stability of O-CD/PVA composites is dramatically improved. All the results confirm that the network of the polymer is suitable for the dispersion of CDs, and the formed full-color CD/PVA films show the desired quench-resistant solid-state fluorescence. These advantages make the full-color CD/PVA films potentially applicable as the color conversion layer for LEDs.

**Fig. 7:**
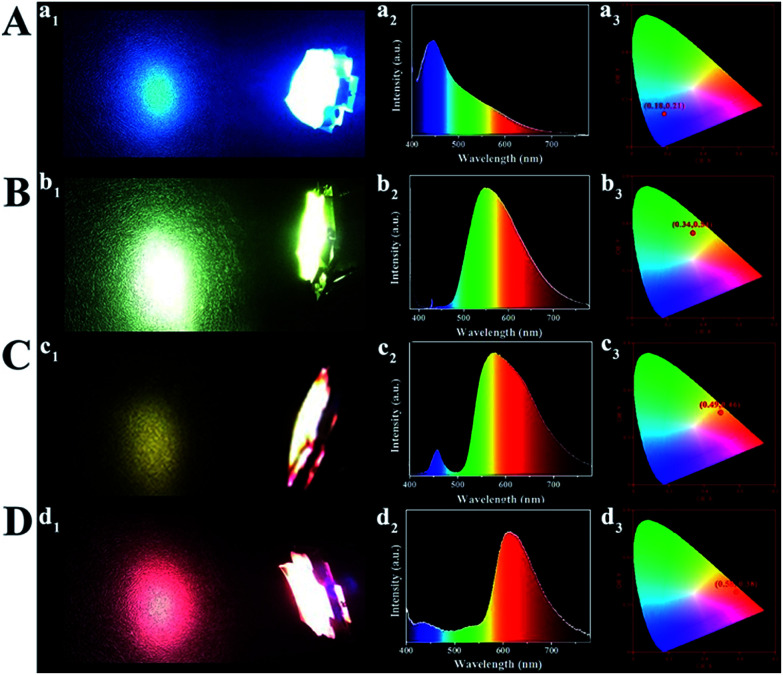
LED prototypes with blue (A), green (B), yellow (C), and orange-red (D) light emitting CD/PVA films as phosphors. (a_1_–d_1_) Fluorescence images; (a_2_–d_2_) EL spectra; (a_3_–d_3_) CIE chromaticity coordinates of the LEDs.

The monochrome blue, green, yellow and orange-red down-conversion LED devices are prepared by coating CD/PVA films on the same chips (emission at 365 nm). As shown in Fig. S24,† at various voltages or currents, the luminous intensity of each color-emitting LED is different. The corresponding EL spectra of light emission are illustrated in Fig. S25,† and each of them is recorded at different device current values. For blue, yellow and orange-red down-conversion LEDs, as the applied current changes, their EL intensity changes, but for the green down-conversion LED, it remains the same. So, it can be inferred that they are promising in practical lighting applications. Fig. 7 presents the LED prototypes with blue (A), green (B), yellow (C), and orange-red (D) light emitting CD/PVA films as phosphors. The four LED devices emit bright light with the Commission Internationale de L'Eclairage 1931 (CIE) coordinates of (0.18, 0.21), (0.34, 0.54), (0.49, 0.46), and (0.58, 0.38), respectively.

**Fig. 8:**
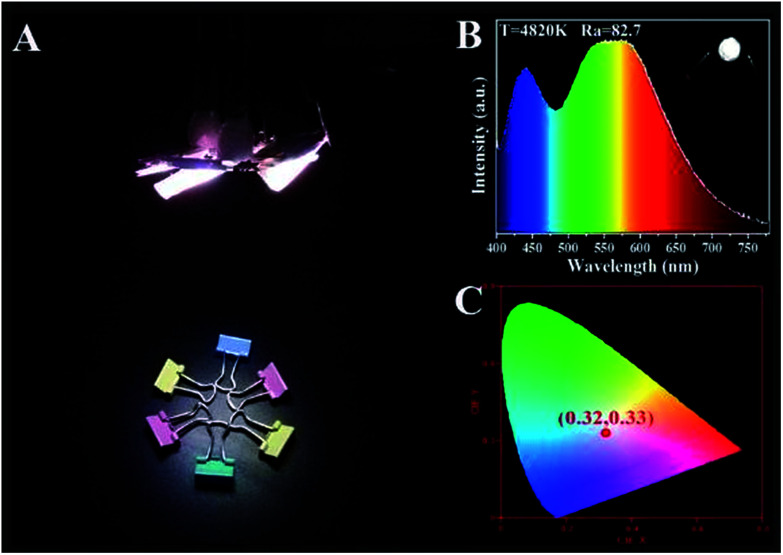
LED prototypes with white light emitting CD/PVA films as phosphors. (A) Fluorescence images; (B) EL spectra; inset: optical image of the LEDs; (C) CIE chromaticity coordinate of the LEDs.

Based on the theory of colorimetry, white light-emitting nanomaterials can be obtained by mixing blue, green, and red light-emitting CDs. By varying the weight ratios of the three CD/PVA films, the white LED with CIE color coordinates of (0.32, 0.33) can be obtained, which approach those of pure white light (0.33, 0.33) (Fig. 8A and C). The relevant EL spectrum is shown in Fig. 8C, which covers most of the visible-light region from 400 to 780 nm.^14^ Moreover, the CCT and CRI of the WLEDs are 4820 K and 82.7, respectively. The light of the as-prepared WLEDs is close to natural sun light.^10^ All the results illustrate that the full-color CDs can meet the requirements of the full wavelength emission of LEDs.

## Conclusions

In summary, the controllable synthesis of CA-based full-color CDs is realized, with the pyrolysis temperature as the control parameter. Two, four, and six color-emitting CD samples are obtained by modifying the reaction temperature from 120 to 180 °C. Detailed characterization results prove that temperature plays an important role in the formation of the surface states of CDs, and surface state directly determines the luminescent properties of CDs. Noticeably the intensity of the typical C=O and N–H stretching vibrations increases, while the strength of O–H stretching vibrations decreases, as one goes from B-CDs_120_ to O-CDs_180_. As the pyrolysis temperature increases, the degree of surface passivation is enhanced and the doping content of heteroatom (*e.g.* N) is increased, which results in the change of composition and structure of the surface state luminophore. The approach used here is amenable to large scale synthesis of CDs for LED applications. CD/PVA and CD/PVP devices are demonstrated. The weight ratios of the three CD/PVA films are optimized to obtain white LEDs with CIE color coordinates of (0.32, 0.33) and a CRI of 82.7.

## Conflicts of interest

There are no conflicts to declare.

## Supplementary Material

NA-001-C8NA00329G-s001
