# Supplementary material for: Temperature-controlled spectral tuning of full-color carbon dots and their strongly fluorescent solid-state polymer composites for light-emitting diodes
Source: Nanoscale Adv. 2019 Jan 17;1(4):1413–20. doi: 10.1039/c8na00329g (PMC9417643; doi:10.1039/c8na00329g)
Supplement: NA-001-C8NA00329G-s001 [file NA-001-C8NA00329G-s001.pdf]

Electronic Supplementary Material (ESI) for Nanoscale Advances.
This journal is © The Royal Society of Chemistry 2019

Supplementary Information

Temperature-controlled spectral tuning of full-color carbon dots and their strongly fluorescent solid-state polymer composites for light-emitting diodes

Tantan Hu,[b] Chan Wang,*[b] Tiju Thomas,[c] Zhuoqi Wen,[a] Chuanxi Wang,*[a] Qijun Song[b] and Minghui Yang*[a]

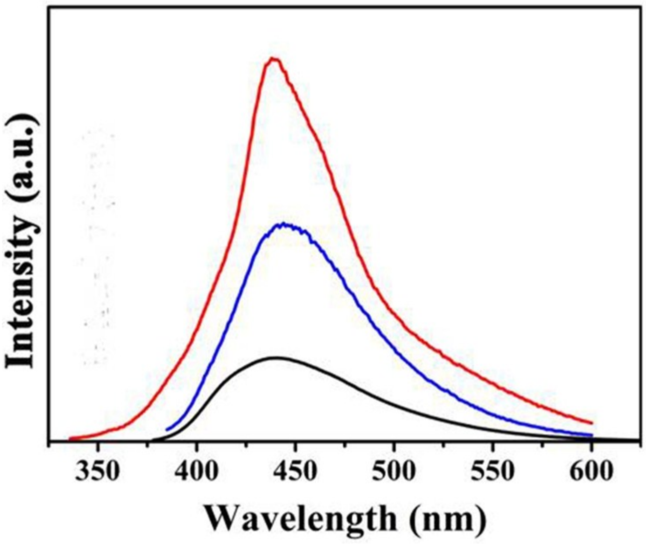

Figure S1. The PL spectra of blue light-emitting CDs prepared at temperature of 120 (black line), 150 (blue line), and 180 ℃ (red line), respectively.

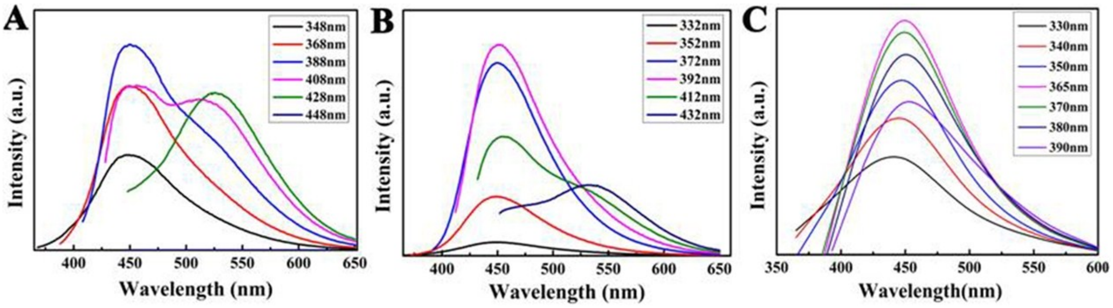

Figure S2. The PL spectra of blue light-emitting CDs prepared at temperature of 120 (A), 150 (B), and 180 ℃ (C), respectively.

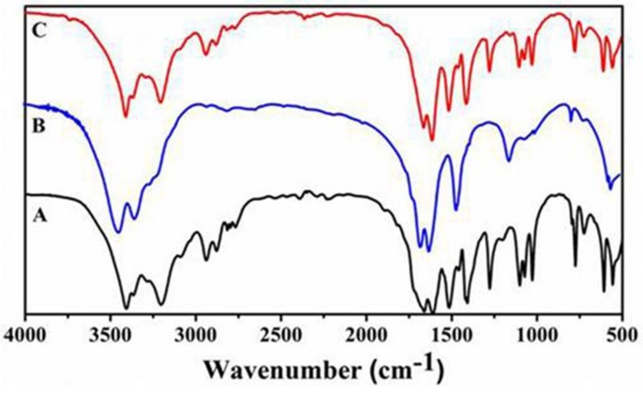

Figure S3. FT−IR spectra of blue light-emitting CDs prepared at temperature of 120 (A), 150 (B), and 180℃ (C), respectively.

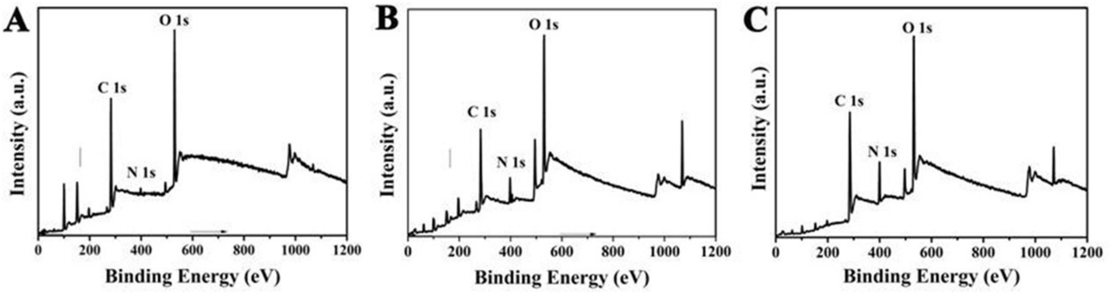

Figure S4. Full-survey XPS spectrum of blue light-emitting CDs prepared at temperature of 120 (A), 150 (B), and 180℃ (C), respectively.

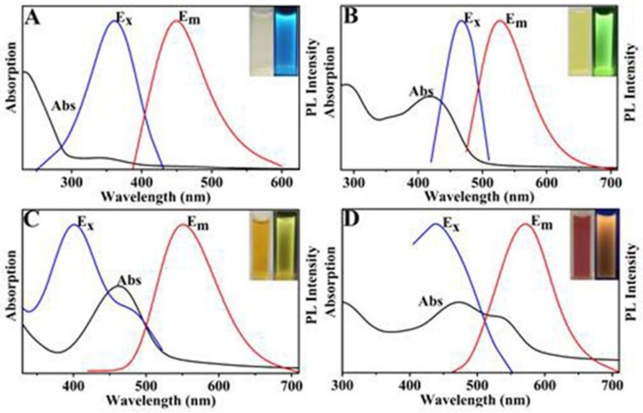

Figure S5. The absorption curves, excitation and emission spectra of B-CDs (A), G-CDs (B), Y-CDs (C) and O-CDs (D), prepared at 180℃, respectively. Inset legends are photographs of the four samples in aqueous solution under daylight (left) and UV light (right).

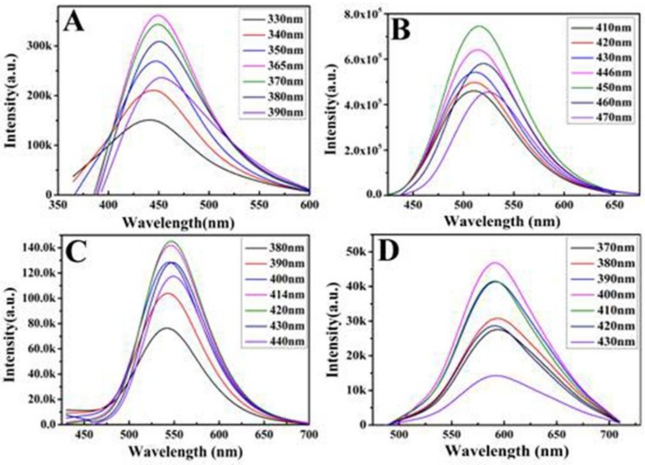

Figure S6. The PL spectra of B-CDs (A), G-CDs (B), Y-CDs (C), and O-CDs (D) with different excitation wavelengths, prepared at 180℃, respectively.

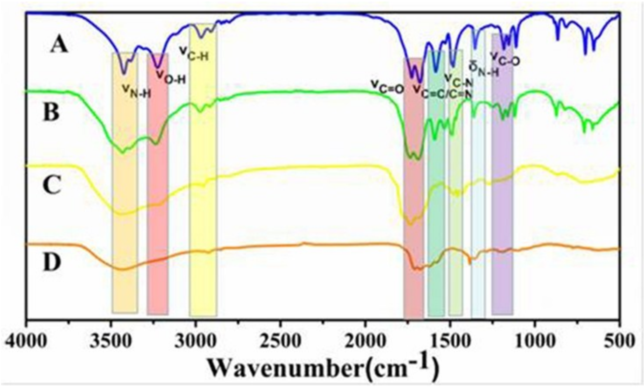

Figure S7. FT−IR spectra of B-CDs (A), G-CDs (B), Y-CDs (C) and O-CDs (D), prepared at 180℃, respectively.

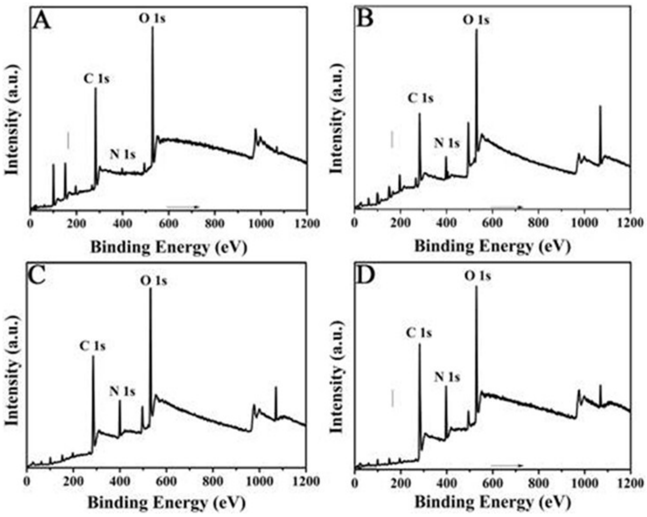

Figure S8. Full-survey XPS of B-CDs (A), G-CDs (B), Y-CDs (C) and O-CDs (D), prepared at 180℃, respectively.

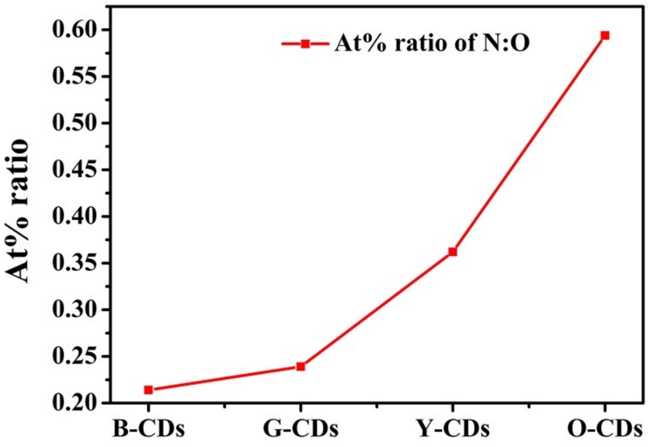

Figure S9. At% ratio of N/O in B-CDs, G-CDs, Y-CDs and O-CDs, prepared at 180℃, respectively.

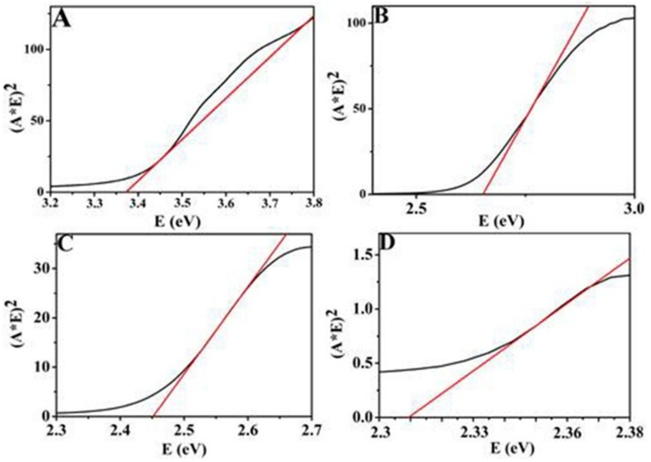

Figure S10. The band-gap energy spectra of B-CDs (A), G-CDs (B), Y-CDs (C), and O-CDs (D), prepared at 180℃, respectively.

Table S1. Band-gap energy data Analyses of four typical CDs, prepared at 180℃.

Sample	Theoretical value (E=1240/λ)	Calculated value	δ(relative error)
**B-CDs**	2.79	3.37	20.8%
**G-CDs**	2.43	2.61	7.4%
**Y-CDs**	2.29	2.45	7.0%
**O-CDs**	2.12	2.31	9.0%

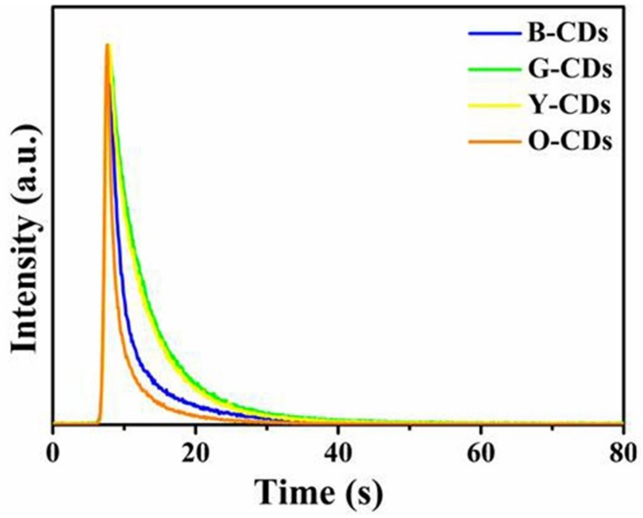

Figure S11. Fluorescence decay profiles of B-CDs, G-CDs, Y-CDs, and O-CDs, prepared at 180℃, respectively.

Table S2. Fluorescent lifetime and relative quantum yield data analyses of the four special CDs, prepared at 180℃, respectively.

Sample	τ_1(ns)	Percent(%)	τ_2(ns)	Percent(%)	τ_{ave}(ns)	QY
**B-CDs**	3.98	56.87	9.66	43.13	6.43	19.4%
**G-CDs**	2.98	31.90	7.23	68.10	5.87	27.8%
**Y-CDs**	1.43	40.05	8.60	59.95	5.73	14.6%
**O-CDs**	0.97	40.59	5.90	59.41	3.90	2.0%

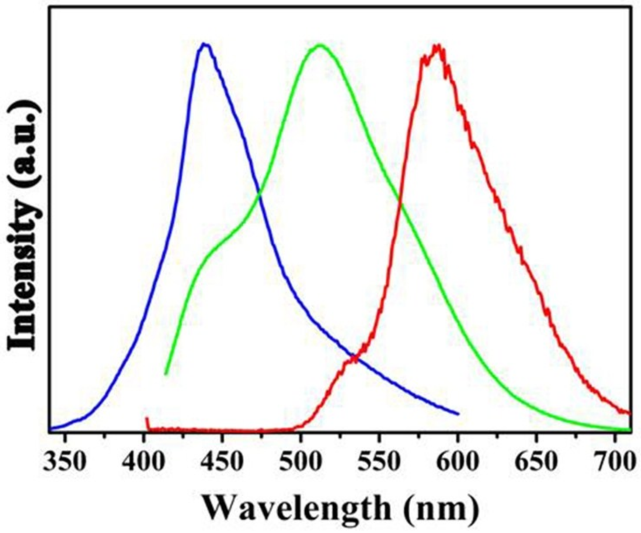

Figure S12. The PL spectra of B-CDs$_{120}$ (blue line), G-CDs$_{150}$ (green line), and O-CDs$_{180}$ (red line), respectively.

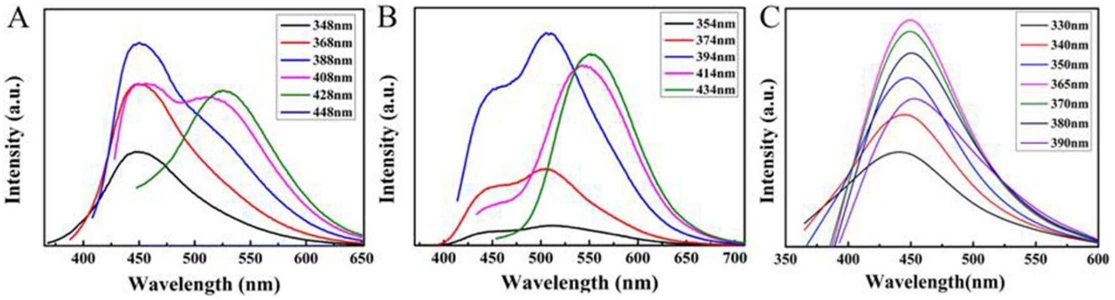

Figure S13. The PL spectra of B-CDs$_{120}$ (A), G-CDs$_{150}$ (B), and O-CDs$_{180}$ (C) with different excitation wavelengths.

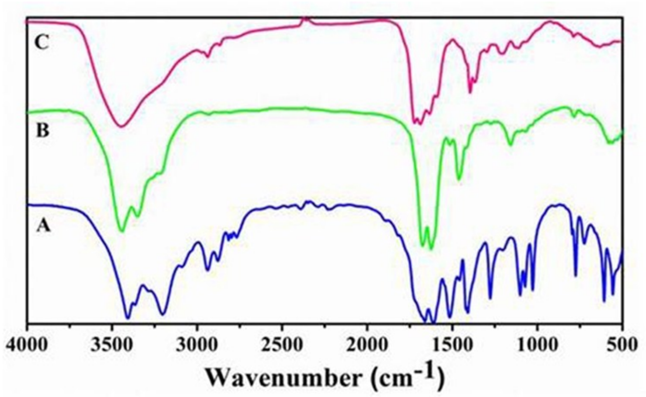

Figure S14. FT–IR spectra of B-CDs$_{120}$ (A), G-CDs$_{150}$ (B), and O-CDs$_{180}$ (C), respectively.

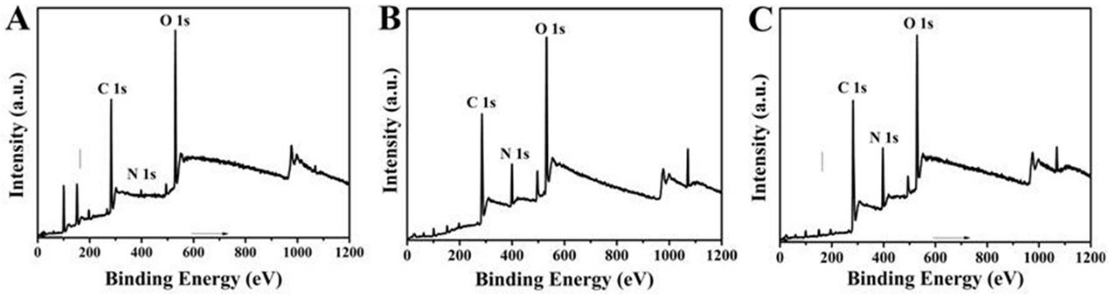

Figure S15. Full-survey XPS spectra of B-CDs$_{120}$ (A), G-CDs$_{150}$ (B), and O-CDs$_{180}$ (C), respectively.

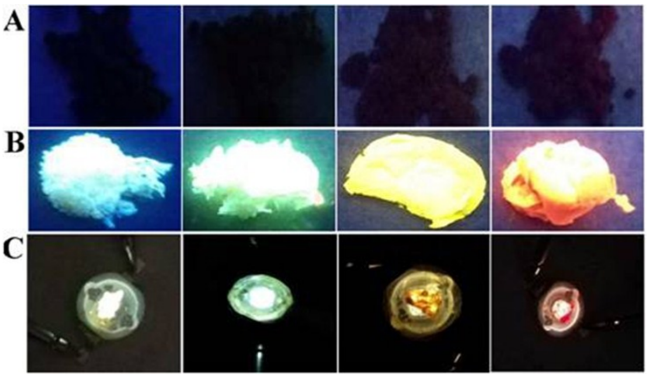

Figure S16. A: the pictures of B-CDs, G-CDs, Y-CDs and O-CDs under 365nm UV light, respectively; B: the images of B-CDs/PVP, G-CDs/PVP, Y-CDs/PVP and O-CDs/PVP under 365nm UV light, respectively; C: the photoes of four LEDs under fixed voltage, with CDs/PVP powder as emitting layers, respectively.

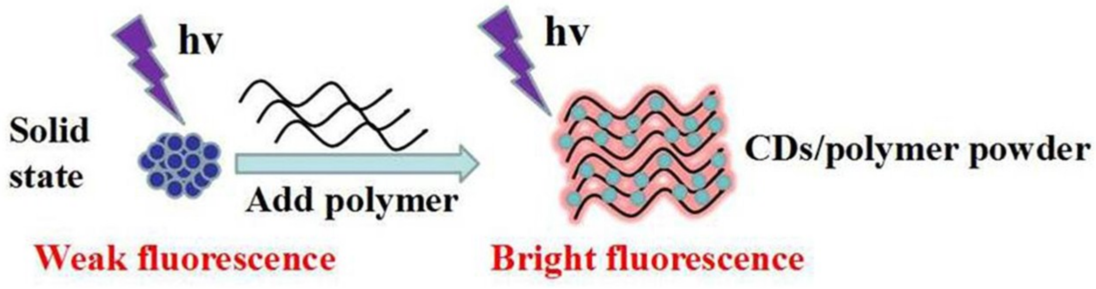

Figure S17. Schematic diagram of the anti-aggregation-induced quenching effect.

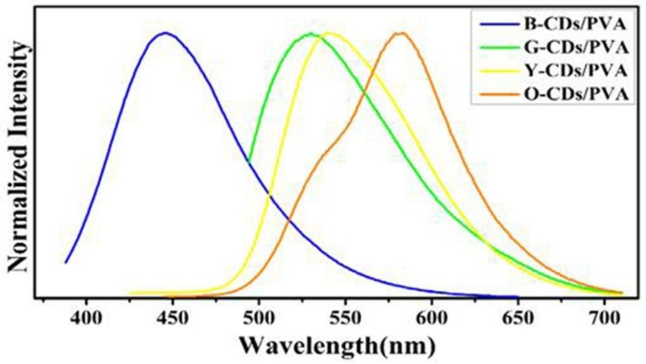

Figure S18. The emission spectra of B-CDs/PVA, G-CDs/PVA, Y-CDs/PVA and O-CDs/PVA films, respectively.

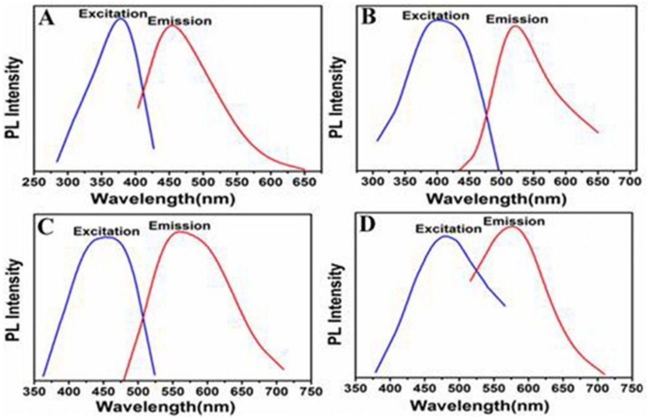

Figure S19. The excitation and emission spectra of B-CDs/PVA (A), G-CDs/PVA (B), Y-CDs/PVA (C) and O-CDs/PVA (D) in solution, respectively.

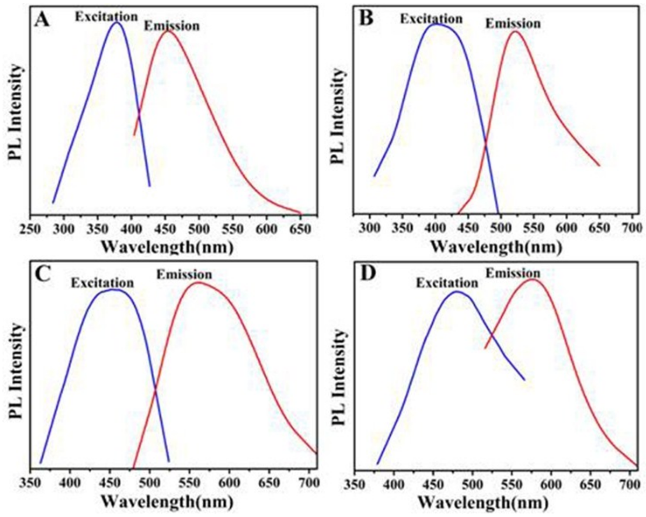

Figure S20. The excitation and emission spectra of B-CDs/PVA film (A), G-CDs/PVA film (B), Y-CDs/PVA film (C) and O-CDs/PVA film (D), respectively.

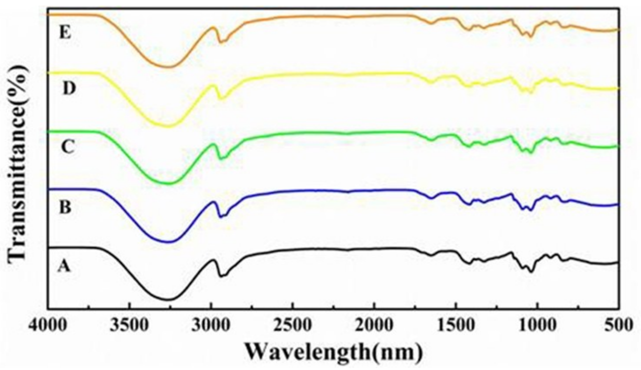

Figure S21. The FTIR spectra of pure PVA film(A), B-CDs/PVA film (B), G-CDs/PVA film (C), Y-CDs/PVA film (D), O-CDs/PVA film (E), respectively.

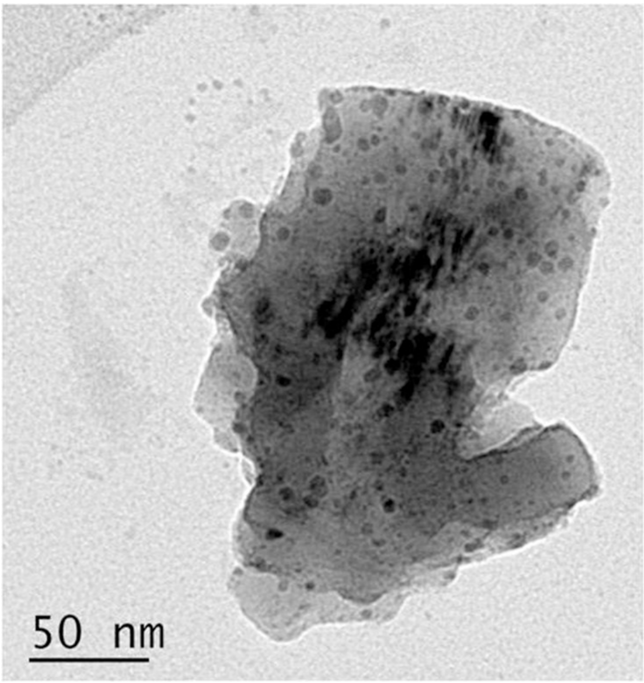

Figure S22. TEM image of CDs/PVA films.

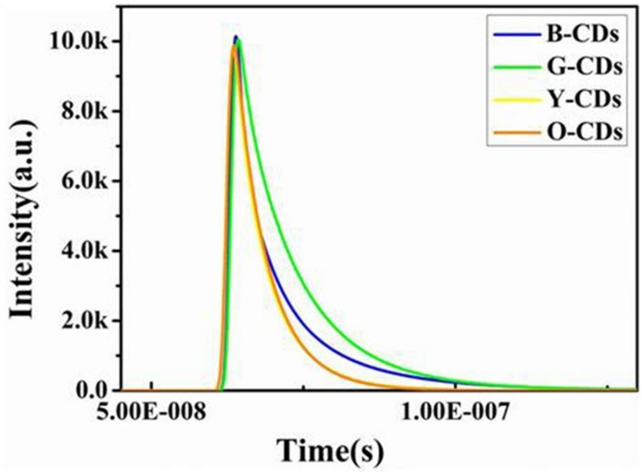

Figure S23. Fluorescence decay profiles of B-CDs/PVA film, G-CDs/PVA film, Y-CDs/PVA film, O-CDs/PVA film, respectively.

Table S3. Fluorescent lifetime and relative quantum yield data analyses of the four CDs/PVA films.

Sample	τ_{ave}(ns)	QY
**B-CDs/PVA**	3.85	5.3%
**G-CDs/PVA**	7.77	12.4%
**Y-CDs/PVA**	4.88	8.9%
**O-CDs/PVA**	2.91	6.9%

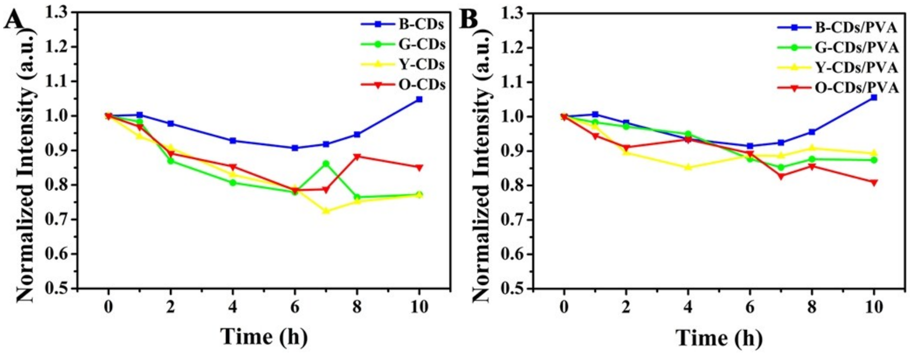

Figure S24. Relative PL intensity of multi-color emissive CDs and CDs/PVA films kept for up to 10h at 365 nm UV light irradiation. A: B-, G-, Y-, and O-CDs; B: B-, G-, Y-, and O-CDs/PVA films.

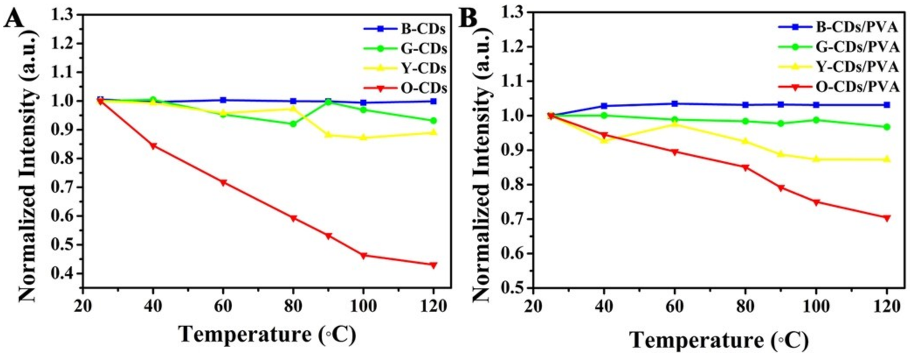

Figure S25. Relative PL intensity of multi-color emissive CDs and CDs/PVA films kept for 1 h under different temperatures as indicated. A: B-, G-, Y-, and O-CDs; B: B-, G-, Y-, and O-CDs/PVA films.

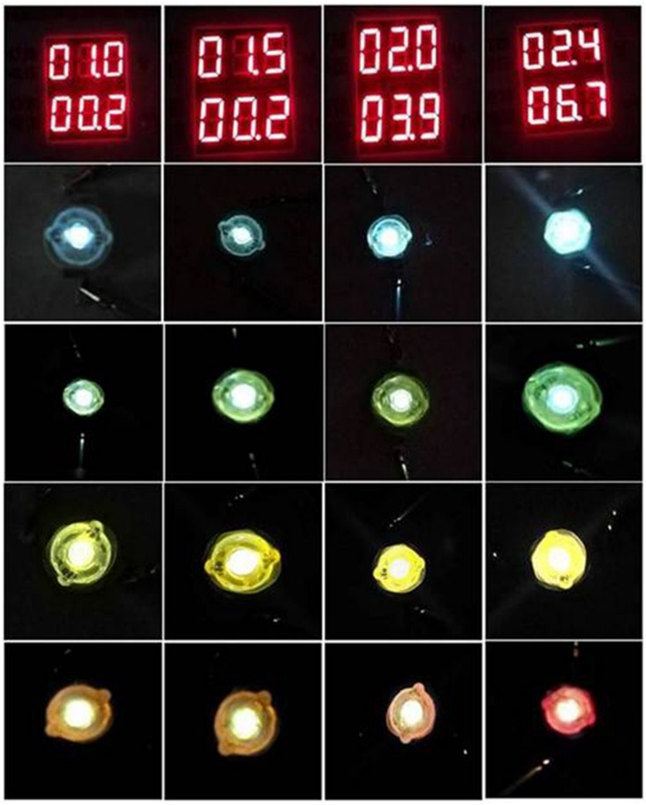

Figure S26. The photoes of four LEDs under different voltage, with CDs/PVA films as emitting layers.

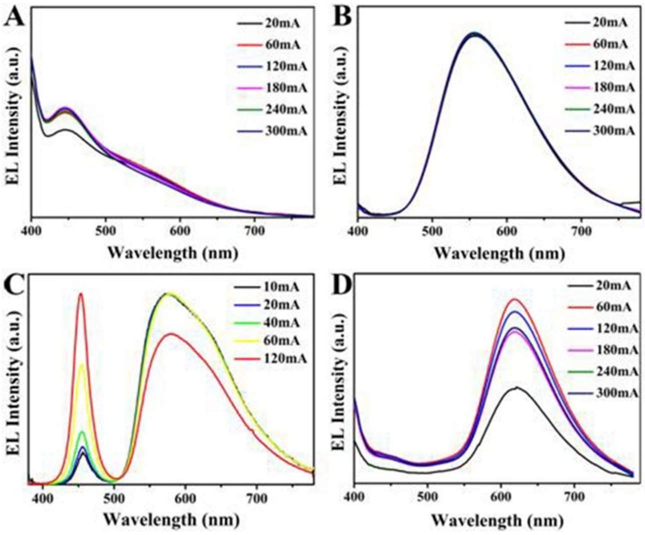

Figure S27. Electroluminescence spectra of four LEDs under different current with B- (A), G- (B), Y- (C), and O-CDs (D)/PVA films as emitting layers.
